# Explain the Experience of Family Caregivers Regarding Care of Alzheimer's Patients: A Qualitative Study

**DOI:** 10.3389/fpsyg.2021.699959

**Published:** 2021-06-24

**Authors:** Hadis Ashrafizadeh, Mahin Gheibizadeh, Maryam Rassouli, Fatemeh Hajibabaee, Shahnaz Rostami

**Affiliations:** ^1^Nursing and Midwifery School, Ahvaz Jundishapur University of Medical Sciences, Ahvaz, Iran; ^2^Nursing Care Research Center in Chronic Diseases, Ahvaz Jundishapur University of Medical Sciences, Ahvaz, Iran; ^3^Cancer Research Center, Shahid Beheshti University of Medical Sciences, Tehran, Iran; ^4^School of Nursing and Midwifery, Tehran University of Medical Sciences, Tehran, Iran

**Keywords:** family cargivers, Alzhieimer's disease, nurse, Iran, conventional content analysis

## Abstract

**Introduction:** Due to the long-term and progressive nature of Alzheimer's disease, these patients need caregivers who will be responsible for their long-term care and who may then experience an increasing burden related to the progressive disease course, so it is important to understand the experiences of caregivers. The aim of this study was to explain the experience of family caregivers regarding care of Alzheimer's patients.

**Methods:** The present qualitative study employed a conventional content analysis approach and was conducted in Iran in 2020. This research was done through in-depth and semi-structured interviews with 11 qualified caregivers enrolled in a purposive sampling method. Interviews continued until data saturation. Data analysis was performed simultaneously with data collection. Interviews were recorded, transcribed and analyzed through Graneheim and Lundman style content analysis and data management was done with MAXQDA software. In order to achieve the accuracy and validity of the study, the Four-Dimensions Criteria (FDC) by Lincoln and Guba, credibility, dependability, confirmability, and transformability were considered and used.

**Results:** A total of 11 caregivers with mean age and standard deviation 48, ±26.12 participated in the study. The acquired data were put in two main categories of “burnout and exhaustion” with six subcategories and “excellence and personal growth” with three subcategories.

**Conclusion:** In this study, we found that perceptions of caregivers' role were not the same among study participants, who experienced both positive and negative dimensions of care provision to AD patients. The care experience has a spectrum that, in some people, leads to positive outcomes such as growth. A major part of caregiver challenges is related to the burden of caregiving strain and the erosive nature of the disease. Therefore, health planners should identify the challenges, pain and suffering of caregivers and seek to address them through appropriate strategies.

## Introduction

Alzheimer's disease, as a chronic and degenerative disease, is turning into a global health priority due to the increase in its incidence and prevalence as well as its impacts on quality of life (Prince et al., 2013; Kordasiewicz et al., 2018). Patients suffering from Alzheimer's disease need others to meet their daily needs, and their dependence increases over time, to the point that 73% of the cases need care more than 40 h a week (Oliva Moreno and Osuna Guerrero, 2009). This disease is also accompanied by high economic expenditures (Gustavsson et al., 2011). Hence, considering its nature, management of this disease is a great challenge for social and health policies in different countries (Moreno-Cámara et al., 2019).

It has been estimated that 83% of care for patients with Alzheimer's disease is carried out using the family-based model (Fujisawa and Colombo, 2009), which is characterized by the great involvement of families in care and lower participation of formal services (del-Pino-Casado et al., 2011). In this context, informal caregivers are the main providers of care services for elderly individuals suffering from Alzheimer's disease at home and take considerable responsibilities in this respect (Bieber et al., 2018). The status and burden of such care services are affected by a variety of factors (Klosek et al., 2012).

Due to the long-term and progressive nature of Alzheimer's disease, these patients need caregivers who accept their care responsibility in the long run, which may result in the incidence of such challenges as physical, emotional, and mental fatigue among caregivers (Guide, 2013). Nonetheless, studies have shown caregivers' different perceptions of their roles. Numerous investigations have demonstrated that informal caregivers suffered from psychological complications, depression, stress, and mental pressure (Chiao et al., 2015; Lindeza et al., 2020). Besides, nearly one-fourth of the caregivers of patients with Alzheimer's disease had anxiety (Mahoney et al., 2005). Overall, the challenges resulted from taking care of patients with Alzheimer's disease are so heavy that family caregivers are usually called invisible secondary patients (Brodaty and Donkin, 2009). On the other hand, some caregivers have a positive attitude toward care conditions, thereby improving the psychological outcomes of care and developing an anti-shock function against negative consequences. In this regard, some caregivers of patients with Alzheimer's disease reported positive experiences, including feeling of satisfaction, feeling of skillfulness and capability, improvement of the quality of relationships with patients, and self-efficacy (Farhadi et al., 2018). Considering the fact that the care construct is multi-dimensional, attention should be paid to all its positive and negative aspects (Lloyd et al., 2016).

Considering the particular features of Alzheimer's disease, caregivers' experiences and attitudes regarding taking care of such patients are complicated and can be affected by various factors (Yektatalab et al., 2012). Due to the impact of sociocultural factors, values, and family traditions on care, caregivers' experiences and attitudes have to be explored in different cultural backgrounds. In Iran, nuclear and extended families are the primary support systems for patients. In other words, strong emotions and relationships exist among family members (Shamsi et al., 2016). Hence, families, specifically closer members, normally have the responsibility to take care of such patients. Due to the lack of formal and informal organizations as well as professionals for training of care, families have to take care of their patients with extremely limited facilities, which doubles these caregivers' challenges (Mohammadi and Babaee, 2011). Since culture and religious teachings rule the care atmosphere in families in Iranian societies and caregivers' experiences and viewpoints are associated with the society's cultural background, developing a deep understanding of family care experiences and the consequent sufferings can help health service providers to plan for educational and support programs compatible with their needs and to design interventions for preventing such challenges (Lund et al., 2014). Thus, the present study aims to determine family caregivers' experiences of taking care of patients with Alzheimer's disease.

## Materials and Methods

### Study Design

In this qualitative study, conventional content analysis was performed to determine family caregivers' experiences of taking care of patients with Alzheimer's disease. This study aimed to describe a phenomenon. This is mainly done in case of paucity of theories or literature regarding the phenomenon under investigation, which causes researchers not to apply the presupposed issues (Hsieh and Shannon, 2005).

### Study Setting

The study participants included the family caregivers of patients suffering from Alzheimer's disease who were selected through purposive sampling by referring to the active branches of the Alzheimer's Association in the capitals of some provinces. The inclusion criteria of the study were being able to speak Persian, aging at least 18 years, being the patient's first-degree relative, having taken care of the patient for at least 6 months, not suffering from preexistent psychological disorders, and being willing to take part in the research and express one's experiences of taking care of a patient with Alzheimer's disease. It should be noted that the time and place of the interviews were arranged with the participants. Totally, 11 participants were interviewed until reaching data saturation; i.e., the point when no new data were achieved and the collected information confirmed the previously gathered points.

### Data Collection Procedure

The study data were collected via semi-structured, in-depth interviews from April to September 2020. Based on the participants' agreement, face-to-face interviews were conducted and recorded using an MP4 recorder. The interviews were begun with the following questions: “can you describe a usual day of yours,” “please talk about your experiences of taking care of elderly patients with Alzheimer's disease,” and “what care services do you need.” Then, the interviews were continued using probing questions, such as “can you explain more” and “can you give an example.” Each interview lasted for 20–45 min. Overall, 13 interviews were conducted with 11 caregivers (three patients were interviewed twice).

### Data Analysis

Data analysis was done simultaneously with data collection. In order to start data analysis, the recorded interviews were played several times and transcribed by the first author. Data management was done using MAXQDA-v13 software (Kuckartz and Rädiker, 2019), and qualitative data analysis was carried out based on the method proposed by Graneheim and Lundman (Graneheim and Lundman, 2004). In doing so, the interviews were transcribed immediately after they were finished. Then, the transcripts were reviewed to obtain an overall understanding. Since qualitative research requires immersion in data, the researcher listened to the interviews several times. After that, an abstract was written for each interview and the hidden meanings were extracted. Then, meaning units were extracted from the participants' words in form of primary codes. These codes were categorized based on meaning and conceptual similarities. It should be noted that the data in all main categories and subcategories followed a descending trend. Finally, the data were classified into main categories and the themes were extracted (Graneheim and Lundman, 2004). An example of data analysis has been presented in Table 1.

**Table 1:** Detailed information about the main categories and subcategories.

**Main categories**	**Subcategories**	**Basic codes**	**Quotation**
Burnout and exhaustion	Physical burnout	Fatigue and exhaustion of caregivers Disruption of the process of caring life Need full-time patient care	“*Sometimes, I am there, my mother is there, my brother is there, but we get tired. We allocate all our time to him/her.”* She continued: “*The people who take care of patients develop a worse status, because they have to allocate all their time to their patients.” (patients' daughter)*
Growth and excellence	Internal satisfaction with care	Feeling happy as a result of caring for the patient	“*At the beginning, I didn't know how to forge a relationship with my patient and be kind. Later, I studied and when I saw that s/he got better with better care provision, I felt happy. The patient's reaction helped me feel better and provide more care services.”*

### Rigor

In order to determine the trustworthiness of the data, use was made of the criteria proposed by Guba and Lincoln (1994). Accordingly, data credibility was determined via prolonged engagement with the research data for 10 months, returning the interview transcripts to the participants and gaining their approval, and assessment of the interview transcripts, codes, and themes by three experts. In addition, dependability was determined by using a combination of methods for data collection (interview, observation, and field notes). In addition, the data were evaluated by an external observer who was familiar with qualitative studies. In order to determine confirmability, all research processes, particularly data collection, data analysis, and formation of categories, were recorded and presented to be used by audiences and readers. Finally, the participants' experiences and characteristics were well-described so as to determine the transferability of the data. Besides, the participants were selected with maximum variation (Schwandt et al., 2007).

### Ethical Consideration

This study was approved by the Ethics Committee of Ahvaz Jundishapur University of Medical Sciences (IR.AJUMS.REC.1398.781, proposal No. NCRCCD-9835). After obtaining the introduction letter from the University, the research team referred to the intended settings in order to identify the eligible participants. The participants were assured about the confidentiality of their information, publication of results, and their right to withdraw from the study. In addition, the participants were provided with nicknames in order to ensure anonymity. Oral and written informed consent was also obtained from the participants.

## Results

The study was conducted on 11 caregivers with the mean age of 48, ±12.26 years. The participants' characteristics have been presented in Table 2. The extracted codes from the interviews resulted in the emergence of two main categories; i.e., “burnout and exhaustion” and “growth and excellence.” Detailed information about the main categories and subcategories has been presented in Table 3.

**Table 2:** Demographic characteristics of the participants in the study.

**Number**	**Age (year)**	**Gender**	**Marital status**	**Level of education**	**Job**	**Ratio with caregiver**
1	48	Female	Married	Diploma	Housewife	Father
2	71	Male	Married	Bachelor	Civil engineer	Spouse
3	35	Female	Married	Bachelor	Housewife	Mother
4	52	Female	Single	PhD	Nurse	Father
5	45	Female	Single	Master seicences	Teacher	Father
6	56	Female	Married	Illiterate	Housewife	Father
7	45	Female	Married	Diploma	Housewife	Father
8	65	Male	Married	Diploma	Housewife	Father
9	35	Female	Married	Diploma	Housewife	Mother
10	33	Female	Married	Illiterate	Housewife	Father
11	43	Female	Married	Illiterate	housewife	Father

**Table 3:** Theme, main categories, and subcategories from the conventional content analysis of the interviews.

**Main categories**	**Subcategories**
Burnout and exhaustion	Mental regression
	Physical burnout
	Role conflict
	Entrusting a family member with the care responsibility
	Neglecting the patient
	Economic burden of the disease
Growth and excellence	Stability and endurance
	Spiritual growth
	Internal satisfaction with care

### Burnout and Exhaustion

One of the main experiences rapidly mentioned by the caregivers was the erosive nature of care for patients with Alzheimer's disease. Such a difficult care should require multidimensional targeted approach. information regarding burnout and exhaustion was then divided into 6 subcategories, namely “mental regression,' “physical burnout,” “role conflict,” “entrusting a family member with the care responsibility,” “neglecting the patient,” and “economic burden of the disease.”

### Mental Regression

The majority of the participants stated that they faced emotional and mental problems, such as depression, hopelessness, feeling guilty, isolation, suffering, stress, tension, irritability, violence, and impatience, which led to negative changes in their families, eventually resulting in their mental regression. In this regard, one of the patients' daughter said:

“*I'm mentally disabled most of the time. I want someone to ask me how I am. Sometimes, people are in particular situations and need a person to talk to, a person who understands their conditions” (*Participant 5*)*.

*Another patient's daughter also mentioned: “Since the disease is long-term, does not improve, and even gets worse, the first thing we experience is stress. When we face the disease over time and accept the disease, we suffer from mental disorders and become depressed” (*Participant 6*)*.

### Physical Burnout

Considering the chronic and degenerative nature of Alzheimer's disease, providing such patients with holistic care is quite a daunting task and leads to physical fatigue. Other related problems include physical injuries, musculoskeletal disorders, sleep disorders, premature aging, inability to transfer patients, fatigue and burnout due to the fast progress of the disease over time, and disruptions in the process of life due to allocating much time to patients for providing them with difficult and long-term care services. In this respect, one of the patients' daughter maintained:

“*Sometimes, I am there, my mother is there, my brother is there, but we get tired. We allocate all our time to him/her.”* She continued: “*The people who take care of patients develop a worse status, because they have to allocate all their time to their patients” (*Participant 1*)*.

### Role Conflict

Role overload occurs when individuals do not have sufficient time and resources for carrying out the commitments associated with their roles. On the other hand, role conflict occurs in case of incompatibility among the expectations from an individual's various roles. According to the participants, role conflict resulted in the incidence of occupational problems. In other words, the main caregivers had to request several days off, were not able to be at work on time, or had to be absent from work. These caused them to be likely to be fired, to have lower income levels, and not to achieve occupational success, all of which enhanced their problems. In this context, one of the patients' daughter stated:

“*My brother doesn't know what to do with his job. He can't go to work. I mean there were several job opportunities in different cities, but he says that he is not able to go, because we have to take care of our father. He has to request days off. Sometimes, he even has to take unpaid leave” (*Participant 10*)*.

### Entrusting a Family Member With the Care Responsibility

Based on the results, one family member was directly responsible for taking care of the patient, while other family members did not cooperate or cooperated to a less extent. Entrusting a family member with the care responsibility resulted in the intensification of care pressure and emergence of further physical, mental, social, and economic problems for the main caregiver. In this respect, one of the patients' daughter said:

“*The most important thing they want is that if there is a patient in a large family, only one member has to take the responsibility for care. Due to their problems, one person is entrusted with the care responsibility. For example, there is a person who has a lonely mother or father. Other members do not cooperate and move away not to get in trouble. So, their problem was related to the care pressure on those families” (*Participant 9*)*.

### Neglecting the Patient

Misbehavior toward patients with Alzheimer's disease refers to an action or lack of an appropriate performance, leading to pain, injuries, and discomfort among elderly patients. In this study, some caregivers conducted some misbehaviors, including lack of accurate follow-up of the treatment process, leaving the patient alone at home, verbal violence when facing repeated questions, not adhering to personal health protocols, losing the patient, and insufficient attention to drugs consumption. One of the patients' daughter mentioned:

“*S/he sometimes asks so many repeated questions and I have shouted at him/her several times. I've got tired” (*Participant 9*)*.

Another patient's daughter also said: “*I*'*m a woman. I can't hold my father and clean him. I have to take him to the bathroom once a week. It is hard for me to transfer him. I can't use adult diapers for him. I use urinary catheters, which has caused numerous problems; he has urinary infection. I can't do it on my own” (*Participant 4*)*.

### Economic Burden of the Disease

All study participants believed that taking care of patients with Alzheimer's disease was very expensive due to the usage of consumable materials (bed sheets, clothes, and disposable materials), non-consumable devices, and necessary equipment, lack of appropriate insurance coverage, cost of medications, and high costs of sending patients to care centers or nursing homes, diagnostic-care services, and such therapeutic methods as home visits by physicians, nurses, physiotherapists, and occupational therapists. It should be noted that some families had low economic levels and, consequently, faced difficulties even for managing their living expenditures. Follow up of patients' treatment and care was also quite costly, which eventually makes caretaking difficult for caregivers and resulted in their psychological harassment. In this regard, one of the patients' daughter maintained:

“*These patients unfortunately have urinary incontinence like children. So, you have to use urinary catheters, which is not possible all the time or you have to use adult diapers, which are costly” (*Participant 7*)*.

Another patient's daughter also stated: “*Costs and economic burdens are imposed on families. Considering their disease stage, patients with Alzheimer's disease have to spend 500-700 thousand Tomans a month to buy medicines” (*Participant 11*)*.

### Growth and Excellence

The study participants referred to some positive aspects and outcomes of care, as well. In other words, they positively expressed their experiences, which had resulted in their growth. This category consisted of three subcategories, namely “stability and endurance,” “spiritual growth,” and “internal satisfaction with care.”

### Stability and Endurance

Stability has been defined as the will power and endurance for resistance against problems. In this study, the caregivers' difficult care conditions led to the incidence of positive personality features like patience, strength against problems, maturity, and increase in their awareness of themselves and the surrounding environment, thereby promoting their understanding. These eventually resulted in the caregivers' compatibility with the challenges associated with taking care of patients with Alzheimer's disease. In this regard, a patient's husband mentioned:

“*When my wife developed the disease, when I referred to healthcare centers, I realized that there were no trainings for me as the caregiver, but I didn't give up. I started studying about the disease and the more I studied, the better the provided care would be. I felt mature. Now, several years have passed. I have helped all caregivers in my city, I have shared all my experiences. I wrote a book, I created a website, I translated many educational clips for caregivers” (*Participant 2*)*.

### Spiritual Growth

The caregivers' encounter with this stressful phenomenon resulted in their spiritual growth. Spiritual growth is a multi-dimensional concept referring to personal relationships with a supreme power, which may result in having a deeper relationship with God, talking to and believing in God, a change in the viewpoint toward life, and deeper thinking about the philosophy of life. Moreover, some participants believed that taking care of such patients was a tool for being examined by God. This viewpoint is derived from the idea that people should surrender and accept the divine providence. Such a permanent relationship with God as well as asking Him for patience and help can ultimately improve caregivers' compatibility with the challenges resulted from care provision. For instance, one of the patients' daughter said:

“*But when I'm calm, I talk to the Creator continually. I have more relationships with God. I see this as a staircase for growth. These are our life courses that we are passing. We haven't been good, so we have to take the course again. When we pass the course, we move forward. If we don't take the courses, we will face more difficult ones. Now we see this in our society, in our families. This disease taught me to realize the value of life” (*Participant 5*)*.

### Internal Satisfaction With Care

While expressing their experiences, some caregivers pointed to satisfaction and happiness. This involved satisfaction with the patients' recovery and tranquility, closeness to parents due to permanent care provision, and parents' thankfulness. In this context, one of the patients' daughter stated:

“*At the beginning, I didn't know how to forge a relationship with my patient and be kind. Later, I studied and when I saw that s/he got better with better care provision, I felt happy. The patient's reaction helped me feel better and provide more care services” (*Participant 3*)*.

## Discussion

In general, caregivers have different perceptions of their role. In other words, the care experience is not necessarily negative, and leads to positive outcomes like growth in some individuals (Lawton et al., 1989). The present study aimed to determine the family caregivers' perceptions of care for patients with Alzheimer's disease. The participants perceived taking care of such patients in both positive and negative dimensions and determination of their experiences resulted in the emergence of two categories, namely “burnout and exhaustion” and “growth and excellence.”

The findings indicated the difficulty of providing care for patients with Alzheimer's disease, leading to physical, mental, familial, financial, and occupational challenges among the caregivers. In fact, caregivers are considered secondary patients, because negative effects exert a huge impact on their health (Sörensen and Conwell, 2011). In the present study, mental regression was among the challenges expressed by the family caregivers. In fact, caregivers were more vulnerable due to stress and care pressure and, consequently, were prone to physical and mental disorders (Jaafari et al., 2016). In this context, Gallagher et al. demonstrated higher care burden and depression among the caregivers of patients with cognitive disorders compared to other caregivers (Gallagher et al., 2011). In addition, depressed caregivers had more unmet needs in comparison to non-depressed ones (Bejjani et al., 2015). Furthermore, caregivers with negative beliefs about their compatibility and those who felt entrapped in their caretaking role had higher complications and depression symptoms and institutionalized the disease soon (Pinquart and Sörensen, 2003), which was in agreement with the results of the present investigation.

Another challenge mentioned in this study was burnout and reduction of physical strength, which was consistent with the results of other studies conducted on the issue. Accordingly, caregivers of patients with Alzheimer's disease experienced worse physical outcomes, including higher levels of stress hormones, compromised immune response, antibodies, higher medication consumption, reduction of cognitive level (Vitaliano et al., 2004), and backache due to patient transfer (Griffiths and Bunrayong, 2016). One of the caregivers' problems was disruptions in their lives due to full-time involvement in patient care. This has been reported to cause various challenges in personal, marital, social, and occupational lives of caregivers (Shafei et al., 2017).

In addition, family caregivers showed low self-efficacy for management of disease-related behaviors due to burnout resulted from their care responsibility (Fortinsky et al., 2002; Cheng et al., 2013; Crellin et al., 2014). These results were in agreement with those of the current research. In some countries, only a limited part of care is carried out by families and the main care services are provided by professional caregivers. Besides, genuine attempts have been made to establish different forms of home- and society-based care services in order to reduce caregivers' care pressure (Low and Fletcher, 2015; Walsh et al., 2020). In Iran, however, due to strong relationships among family members, beliefs and religious values of the family with emphasis on caring for the elderly (Yektatalab et al., 2012), lack of home care centers (Mohamadi Shahbalaghi, 2006), and lack of healthcare services provision by formal and professional caregivers, family caregivers are completely responsible for taking care of patients with Alzheimer's disease (Mohammadi et al., 2011). Thus, a higher care burden is imposed on families and caregivers have to provide difficult care services (Mohammadi and Babaee, 2011). This necessitates families' further attention and support for the main caregiver.

Financial problems were among the caregivers' experiences in the present investigation. In other words, the caregivers encountered numerous challenges due to the financial pressure resulted from taking care of their patients. Evidence has also indicated that caregivers of patients with Alzheimer's disease suffered from a wide range of challenges, including financial problems (Goodall and Harrison, 2008; McCabe et al., 2016; Shafei et al., 2017). Generally, caretaking affects families financially, because caregivers of elderly people with Alzheimer's disease do not have the ability to earn a living. Yet, they need money for their treatment, transfer to hospital, and daily lives, which causes numerous problems (Griffiths and Bunrayong, 2016).

Role conflict was another experience mentioned by the caregivers. In fact, family caregivers have different responsibilities, which results in the loss of their independence and time for management of their roles (Grant et al., 2004). Moghimi (2007) also revealed role conflict as one of the main challenges amongst caregivers. Accordingly, caregivers solely paid attention to their caretaking responsibility, which reduced their participation in their previous occupations, roles, and daily activities (Moghimi, 2007). In other words, caretaking interfered with other responsibilities, such as familial, occupational, and homemaking roles, and resulted in burnout, eventually exposing caregivers to chronic physical and mental problems (Jaafari et al., 2016).

In the current study, one of the caregivers' experiences was lack of cooperation on the part of other family members, which caused them to face more challenges. Although a main caregiver normally manages the care process for patients with Alzheimer's disease, caretaking would be quite difficult and even impossible without others' cooperation (Mohammadi et al., 2011). This can be explained by studying the patterns of community-based care for the elderly, in experienced countries, with the establishment of various forms of home and community care services, efforts have been made to reduce the burden of caring for families, but in Iran, this important issue is left only to the family (Zohari et al., 2006; Mohammadi et al., 2011).

The caregivers under the present investigation mentioned neglecting patients as one of their experiences. Accordingly, misbehaviors and negligence led to further problems for patients, which made the care process more difficult for the family caregivers. In this context, several studies have indicated that caregivers' isolation and care burden resulted in misbehaviors toward patients with Alzheimer's disease (Vida et al., 2002; Vandeweerd et al., 2013). This cognitive disorder was also a major risk factor for misbehaviors toward elderly patients suffering from Alzheimer's disease (Shugarman et al., 2003). Moreover, caregivers' psychological pathology showed that emotional and mental tensions led to negligence and misbehaviors toward patients (Shugarman et al., 2003; Fulmer et al., 2005; Vandeweerd et al., 2013).

Although the family in Iran is an important source of power, support and security at all times, but due to the severe nature of the disease, long-term care of the Alzheimer's patient, related problems, inability to solve these problems due to lack of financial and social support for the health care system and lack of cooperation of other family members on the other hand, increases the overwhelming pressure on caregivers and prevents them from leading a normal life. The results of some studies have shown, due to numerous problems caused by exhausting and long-term care, patient support decreased and increased the neglect of the patient and consequently the suffering of caregivers (Mohammadi et al., 2011; Yektatalab et al., 2012; Nemati et al., 2018).

In contrast to burnout, the other main category extracted in this study was the caregivers' growth and excellence, which was in fact the positive aspect of the participants' evaluation of their care services. The positive experience of care does not have a single meaning, and has been perceived as satisfaction with care, care reward, care experience, and impact on caregiver's self-esteem by different researchers (López et al., 2005; Cohen-Mansfield et al., 2015). It has been reported that caretaking could result in positive changes in people's lives, thereby leading to the feeling of satisfaction (Kate et al., 2012). Some studies have also demonstrated that caregivers felt happy, satisfied, and close to their parents by taking care of patients with Alzheimer's disease (Yamamoto-Mitani et al., 2003; Kate et al., 2012). These results were in line with those of the present research.

Another positive experience expressed by the caregivers was spiritual growth or excellence. Spiritual growth, as the main dimension of post-traumatic growth, could enhance individuals' strength and help them realize the value of life (Hodge and Sun, 2012). In the present study, the caregivers believed that their internal power originated from God's power, which was strengthened through cognition of themselves and the Creator, ultimately leading to better compatibility with challenges. Furthermore, some participants stated that taking care of patients with Alzheimer's disease provided them with the opportunity for active coping and growth, eventually increasing their faith and changing their lives. In the study performed by Lee et al., caregivers of patients with dementia at the end stage of life reported maturity as a positive experience of care. This dimension involved appreciation of life and better relationships with others (Lee et al., 2007). Excellence and personal growth were also the themes disclosed by Farhadi et al. (2018) and Navab et al. (2012), respectively. Accordingly, spiritual growth was achieved when caregivers found the meaning of life and realized the value of their belongings (Navab et al., 2012). Similarly, Tarlow et al. maintained that achievement of the meaning of life, positive direction in life, and realizing the value of life were the major dimensions of patient care (Tarlow et al., 2004).

Another positive experience extracted in the current study was stability and endurance resulted from taking care of patients with Alzheimer's disease. Prior studies indicated that empowerment and promotion of self-confidence and perseverance while facing problems were the main index of post-traumatic growth (Chun and Lee, 2008). In other words, individuals with higher stability and resilience were able to overcome the difficulties of life, achieve better health and independence, become compatible with environmental changes, and recover after the elimination of stressors (Karimirad et al., 2018). Netoo et al. also stated that taking care of elderly patients with Alzheimer's disease could lead to positive achievements, such as increased self-awareness, better compatibility with conditions, self-belief, strength, and kindness (Netto et al., 2009). Although the family caregivers' positive experiences were somewhat similar to those reported in some studies conducted in western countries, but in some ways they were specific to the Iranian society and culture. In Islamic literature, it has been mostly mentioned that hardships and disasters can lead to human growth and increase the stability and endurance of people. Given that the predominant religion of the people in Iran is Islam, religious beliefs will play an important role in dealing with stressful events. In the study of Heidarzadeh et al. (2018), the score of spirituality subscale in Iranian patients was higher than other countries. Therefore, they can be used by researchers in order to decrease the negative care outcomes

Caregivers' perceptions of care are influenced by various individual, social, and cultural factors such as the demographic characteristics of the caregiver and the Older Adults, the demand for care, the prevailing culture in the community, and coping strategies (Farhadi et al., 2016). Although in a few studies, the relationship between the positive and negative aspects of caregivers' perceptions with the duration of the patient's illness, occupation, gender and economic status of the caregiver, the kinship with the patient, the duration of care, and the coexistence with the elderly has been investigated (Aboozadeh Gotabi et al., 2016; Farhadi et al., 2016), but due to the qualitative nature of the present study, it was not possible to assess such relationships. Therefore, it is recommended that this issue be considered in future studies.

### Limitations

This study was conducted on limited groups of the Iranian population, while there are numerous subcultures in the country that are different with regard to culture, lifestyle, race, and language. In order to prevent the impact of this problem, the participants were selected from different provinces with a variety of cultural backgrounds. Furthermore, although the study results revealed the caregivers' experiences, they might not be generalizable due to the qualitative nature of the research. Additionally, data regarding patients' Alzheimer's disease stage or clinical presentation were not reported, therefore, it needs to be considered in future research.

Another limitation of the present study was that most caregivers of children and only one spouse entered the study as a caregiver. Perhaps the reason for this is that the patient's spouse is somehow also an elderly person and due to a variety of underlying diseases are not able to play the main role of caregiver. In such situations, if the spouse is caring, they become much more vulnerable due to stress and the burden of caring and are more at risk of various physical and mental disorders (Jaafari et al., 2016). Different societies have different values, and the difference between societies is the same values, people's attitudes. The values of our country are also a mixture of traditional Iranian values and Islamic values. Attitudes about the status of the elderly are also important given the traditional and religious culture of Iranian families (Jafari, 2018). As a result, according to the religious culture of the Iranian society, the children had a duty to take care of their parents in case of illness.

## Conclusion

Considering Iran's cultural and religious background, family caregivers are considered the main services providers for patients suffering from Alzheimer's disease. In the present study, the caregivers perceived care negatively as “burnout and exhaustion” and positively as “growth and excellence.” Identification and understanding of caregivers' experiences is of particular importance for providing them with services to reduce their pain, suffering, and care burden and to increase their quality of life. It can also help health planners prepare facilities and services for caregivers. Thus, programs should be developed and evaluated in different studies. Moreover, health policymakers are recommended to prepare educational and support packages to eliminate caregivers' challenges and decrease their care pressure through providing patients with formal care services.

## Data Availability Statement

The original contributions presented in the study are included in the article/supplementary material, further inquiries can be directed to the corresponding author.

## Ethics Statement

This study was approved by the Ethics Committee of Ahvaz Jundishapur University of Medical Sciences (IR.AJUMS.REC.1398.781, proposal No. NCRCCD-9835). The patients/participants provided their written informed consent to participate in this study.

## Author Contributions

MG, HA, MR, SR, and FH involved in the study conception/design and contributed to the data collection/analysis. MG, HA, MR, and FH drafted the manuscript. MH, HA, and MR in critical revisions for important intellectual content and administrative/technical support and supervised the work. All authors contributed to the article and approved the submitted version.

## Conflict of Interest

The authors declare that the research was conducted in the absence of any commercial or financial relationships that could be construed as a potential conflict of interest.
